# Detection of quantitative trait loci associated with drought tolerance in St. Augustinegrass

**DOI:** 10.1371/journal.pone.0224620

**Published:** 2019-10-31

**Authors:** Xingwang Yu, Jessica M. Brown, Sydney E. Graham, Esdras M. Carbajal, Maria C. Zuleta, Susana R. Milla-Lewis

**Affiliations:** Department of Crop and Soil Sciences, North Carolina State University, Raleigh, North Carolina, United States of America; Institute of Genetics and Developmental Biology Chinese Academy of Sciences, CHINA

## Abstract

St. Augustinegrass (*Stenotaphrum secundatum*) is a warm-season grass species commonly utilized as turf in the southeastern US. Improvement in the drought tolerance of St. Augustinegrass has significant value within the turfgrass industry. Detecting quantitative trait loci (QTL) associated with drought tolerance will allow for advanced breeding strategies to identify St. Augustinegrass germplasm with improved performance for this trait. A multi-year and multi-environment study was performed to identify QTL in a ‘Raleigh’ x ‘Seville’ mapping population segregating for phenotypic traits associated with drought tolerance. Phenotypic data was collected from a field trial and a two-year greenhouse study, which included relative water content (RWC), chlorophyll content (CHC), leaf firing (LF), leaf wilting (LW), green cover (GC) and normalized difference vegetative index (NDVI). Significant phenotypic variance was observed and a total of 70 QTL were detected for all traits. A genomic region on linkage group R6 simultaneously harbored QTL for RWC, LF and LW in different experiments. In addition, overlapping QTL for GC, LF, LW and NDVI were found on linkage groups R1, R5, R7 and S2. Sequence alignment analysis revealed several drought response genes within these regions. The QTL identified in this study have potential to be used in the future to identify genes associated with drought tolerance and for use in marker-assisted breeding.

## Introduction

St. Augustinegrass (*Stenotaphrum secundatum* [Walt.] Kuntze) (*2n* = *2X* = 18) is a warm-season turfgrass that is well adapted to tropical and subtropical regions of the world [1]. It has been a popular turfgrass in the southern United States for its broad leaf blades and rapid stolon elongation, which makes the grass well-suited for sod production [2]. However, one of the greatest challenges that turfgrass industry facing is the limited availability and reduced quality of water for irrigating turfgrass areas. To address these concerns, there is a need to develop St. Augustinegrass cultivars with the improved levels of drought tolerance. Considerable efforts have been devoted to determining the drought response of St. Augustinegrass, including turf quality, leaf firing, percent green cover, canopy temperature and root characteristics [3, 4]. However, the genetic mechanisms controlling these physiological responses are largely unknown.

Plants display a variety of physiological and biochemical responses at the cellular and whole-organism level when under drought stress [5]. Previous studies showed drought tolerance is a complex quantitative trait, which is controlled by multiple small effect genes [6, 7, 8, 9]. Identification of quantitative trait loci (QTL) has been considered as a primary method used to identify molecular markers associated with drought related traits. Merewitz et al. [8] identified QTL associated with drought related traits such as turf quality (TQ), relative water content (RWC) and the normalized difference vegetative index (NDVI) in a creeping bentgrass population. More recently, several QTL associated with leaf water content (LWC), leaf wilting (LW) and CHC under well-watered and drought conditions were found in wild grass *Brachypodium distachyon* [9]. To date, besides cool-season grasses, QTL analysis for drought tolerance has not been widely reported for turfgrass species.

Generation of genetic maps and QTL analysis of desired traits can later be used in marker-assisted selection, to identify the genes underlying the QTL, or for analysis of genomic synteny with related grass species [10]. However, lack of high-density linkage maps has hindered QTL analysis in most turfgrass species. In recent years, advances in high-throughput sequencing technology have provided powerful genotyping tools to develop large numbers of SNP markers, which allowed QTL mapping to be more successful in turfgrass [11, 12, 13, 14]. Using the genotype-by-sequencing (GBS) approach, Yu et al. [14] developed high density linkage maps containing 2871 SNP markers from a ‘Raleigh x Seville’ biparental population in St. Augustinegrass, enabling QTL mapping for traits of interest in this population, including turf quality, leaf texture, genetic color, and turf density. Previous pilot studies on drought tolerance screening found that ‘Raleigh x Seville’ population segregated for drought tolerance. Further mapping of QTL associated with drought traits may shed light on the genetic control of drought tolerance and potential application in marker-assisted selection in St. Augustinegrass breeding. The objectives of this study include (i) evaluating variance of drought related traits of ‘Raleigh x Seville’ population in both greenhouse and field conditions and (ii) identifying QTL associated with drought related traits and mining candidate genes within QTL.

## Materials and methods

### Plant materials and linkage maps

A St. Augustinegrass mapping population was developed by Kimball et al. [15]. This pseudo-F_2_ population containing 115 hybrids was derived from a cross of cultivars ‘Raleigh’ and ‘Seville’. The linkage maps derived from ‘Raleigh x Seville’ population were developed by Yu et al. [14]. The maps were created for each parental genotype and contained nine linkage groups for each parent, named R1-R9 for Raleigh map and S1-S9 for Seville map, which correspond to the nine base chromosome number for diploid St. Augustinegrass [14]. The population was used for phenotypical and physiological evaluation of drought tolerance traits in three different trials under two different environments including two greenhouse trials in 2017 and 2018 (GH17 and GH18), and a field trial at the Sandhills Research Station (Jackson Springs, NC) in 2018 (SRS18).

### Greenhouse experiments

**Fig 1:**
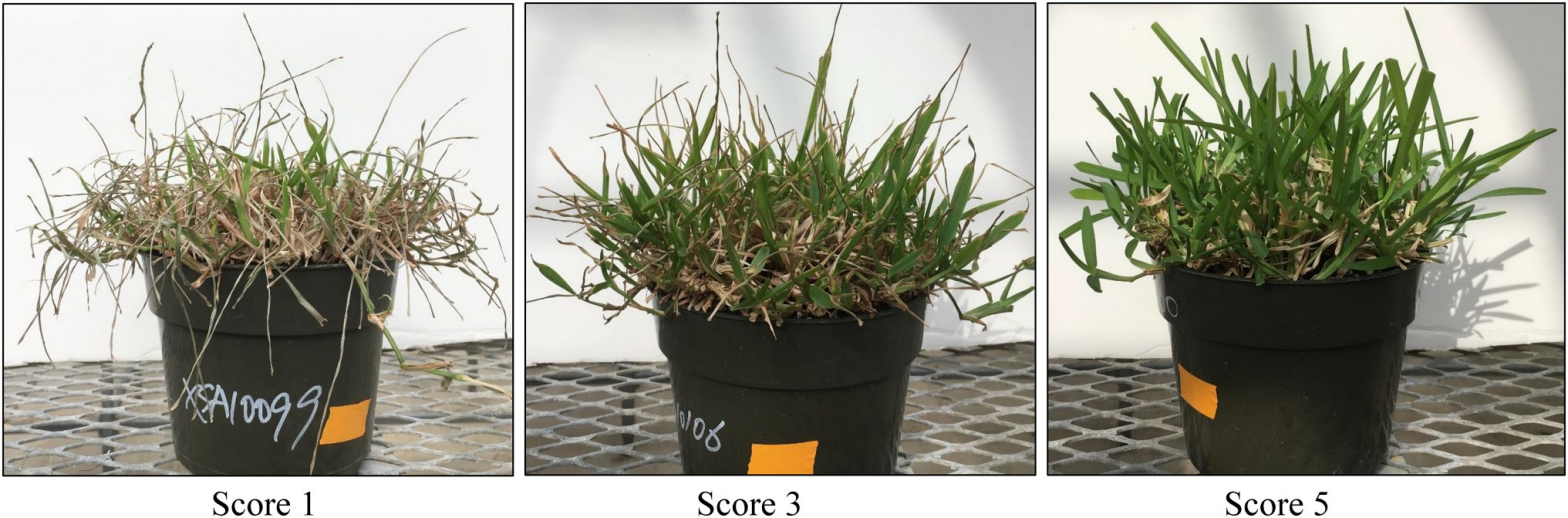
Visual rating of leaf firing score (1–5) on St. Augustinegrass Raleigh x Seville F_1_ population during drought stress in greenhouse trails.

Plants were vegetatively propagated in 15 cm diameter by 11 cm deep pots filled with a mix of sand and Fafard potting mix (Conrad Fafard Inc, Agawam, MA). Plants were established for six weeks before drought treatment in order to allow sufficient growth to form a uniform canopy. Drought stress was applied by withholding water starting on 2 September 2017 for GH17 and on 2 January 2018 for GH18 until soil water content reached approximately 5%, which occurred on 10^th^ day for GH17 and 14^th^ day for GH18. Soil water content was monitored using a TDR 100 Soil Moisture Meter (Spectrum Technologies, Inc. IL). At the end of the drought stress treatment, leaf wilting (LW) and leaf firing (LF) were visually scored on a scale of 1 (no wilting/firing) to 5 (severe wilting/firing) according to National Turfgrass Evaluation Program (NTEP) guidelines (Fig 1) [16]. Leaf relative water content (RWC) was determined according to the formula: RWC = (FW-DW)/(TW-DW) × 100, where FW is fresh weight, DW is dry weight and TW is turgid weight. Relative chlorophyll content (CHC) in leaf was measured using MultispeQ v2.0 (PhotosynQ Inc, East Lansing, MI). In addition, percent green cover (GC) was calculated using digital image analysis with ImageJ software on third day after recovering from stress [17].

### Field experiments

A field trail was planted in June 2016 at the Sandhills Research Station. Plots were 0.91 m × 0.91 m in size. Plots were irrigated and fertilized according to recommended practices for NC and mowed weekly at a 6.35 cm height. Drought stress was applied during the annually recurring drought period for the region. Data were not collected in summer of 2017, as turfgrass were not stressed due to short continuous drought period. After drought stress started on 12 August 2018 and lasted 18 days, visual ratings were taken for leaf firing (LF) using a scale of 1 (no firing) to 5 (severe firing) according to NTEP guidelines [16]. Normal Difference Vegetation Index (NDVI) were assessed using a TCM 500 NDVI Turf Color Meter (Spectrum Technologies, Inc, Aurora, IL).

### Experimental design and statistical analysis

Both greenhouse and field experiments were arranged in a randomized complete block design with three replicates. Analysis of variance (ANOVA) was performed using SAS PROC GLM procedure (SAS Institute, Cary, NC) with replication as random effect. Correlation analysis between traits and trails was performed using SAS PROC CORR procedure (SAS Institute, Cary, NC).

### QTL detection

QTL analysis was performed using the integrated two-way pseudo-testcross approach with MapQTL 6.0 [18]. This approach was applied by analyzing data for each parental meiosis separately. Kruskal-Wallis (KW) analysis, interval mapping (IM) and multiple QTL method (MQM) analysis were performed to detect significant associations between markers and phenotypic traits using a regression approach. Genome-wide LOD thresholds (*p* < 0.05) were determined for each trait using a permutation test with 10,000 iterations. Regions with a LOD score above threshold values were considered as potential QTL intervals. Lastly, the sequences flanking SNP markers that fall within the identified regions of interest were searched against the NCBI NR database using blastn/blastp tools to obtain their orthologs. Gene annotation was conducted using the UniProt database to predict gene function in the QTL regions.

## Results

### Phenotypic trait variation and correlation under drought stress

**Fig 2:**
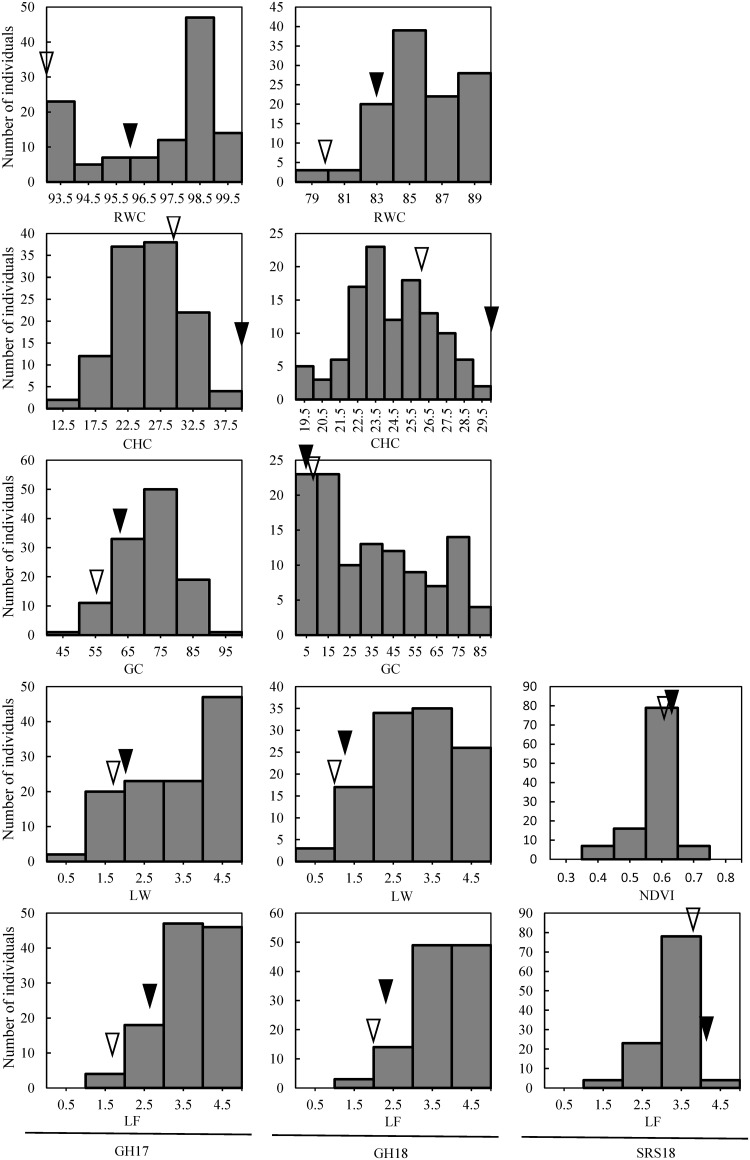
Distribution of chlorophyll content (CHC), percent green cover (GC), leaf relative water content (RWC), leaf wilting (LW), leaf firing (LF) and normalized difference vegetation index (NDVI) values for a St. Augustinegrass Raleigh x Seville F1 population evaluated for drought response under greenhouse (GH17 and GH18) and field conditions (SRS18). Solid triangle indicates Raleigh and white triangle indicates Seville.

The hybrids in the ‘Raleigh x Seville’ population showed a wide range of phenotypical variation in all evaluated traits and all independent trails (Table 1, S1 Table, Fig 2). Values for CHC, GC, RWC, LW and LF ranged from 12.33 to 38.80, 46.10 to 91.80, 77.72 to 99.49, 1.00 to 5.00 and 1.33 to 5.00, respectively, in experiment GH17, and ranged from 19.00 to 29.97, 2.15 to 86.29, 79.01 to 89.31, 1.00 to 5.00 and 1.67 to 5.00, respectively, in experiment GH18. In the field experiment (SRS18), values for NDVI and LF ranged from 0.31 to 0.64 and 1.25 to 4.38, respectively. Genotype effects were observed for all traits in all trails (<0.0001). Significant trail effects were found in RWC, GC, CHC and LF, while genotype-by-trail effects only were found in GC and LF (Table 1 and S1 Table).

**Table 1:** Phenotypic data and genotype variance on chlorophyll content (CHC), percent green cover (GC), leaf relative water content (RWC), leaf wilting (LW), leaf firing (LF) and normalized difference vegetation index (NDVI) for a St. Augustinegrass Raleigh x Seville F_1_ population evaluated for drought response under greenhouse (GH17 and GH18) and field conditions (SRS18).

	GH17					GH18					SRS18	
	CHC	GC	RWC	LW	LF	CHC	GC	RWC	LW	LF	NDVI	LF
**Raleigh**	40.14	62.03	95.60	2.00	2.67	30.55	5.11	82.99	1.33	2.33	0.61	4.08
**Seville**	28.46	54.76	86.87	1.67	1.67	26.31	7.61	79.64	1.00	2.00	0.6	3.83
**Progeny Mean**	25.67	71.43	95.85	3.55	3.86	24.46	35.02	85.66	3.28	3.95	0.53	3.19
**Progeny Min**	12.33	46.10	77.72	1.00	1.33	19.00	2.15	79.01	1.00	1.67	0.31	1.25
**Progeny Max**	38.80	91.80	99.49	5.00	5.00	29.97	86.29	89.31	5.00	5.00	0.64	4.38
**Variance (genotype)**	***	***	***	***	***	***	***	***	***	***	***	***

*** Significance at p-value < 0.001.

No significant correlations among traits were observed between greenhouse and field experiments. However, significant correlations were found between traits under the same conditions. In experiment SRS18, a positive correlation was identified between NDVI and LF (Table 2). In greenhouse evaluations, significant positive correlations (p-value < 0.01) were observed among GC, RWC, LF and LW (Table 2). CHC only showed positive correlations with LW (p-value < 0.01) and LF (p-value < 0.05), but did not with RWC and GC (Table 2). In addition, the correlation analysis between GH17 and GH18 showed all traits have positive correlation between two experiments except LW (S2 Table).

**Table 2:** Correlation coefficients among chlorophyll content (CHC), green cover percent (GC), leaf relative water content (RWC), leaf wilting (LW), leaf firing (LF) and normalized difference vegetation index (NDVI) for a St. Augustinegrass Raleigh x Seville F_1_ population evaluated for drought response under greenhouse and field conditions.

		**Field**		**Greenhouse**†			
	**Trait**	**NDVI**	**LF**	**CHC**	**GC**	**RWC**	**LW**
**Field**	**NDVI**						
	**LF**	0.8958**					
**Greenhouse**	**CHC**	0.0008	0.0027				
	**GC**	0.0197	0.0207	0.0205			
	**RWC**	1.16E-05	2.33E-05	0.0068	0.2736**		
	**LW**	0.0044	0.0025	0.0986**	0.4140**	0.5098**	
	**GH-LF**	0.0037	0.0017	0.0841*	0.4069**	0.4262**	0.7147**

^†^ Greenhouse data averaged from GH17 and GH18.

* and ** significance at p-value < 0.05 and 0.01, respectively.

### QTL detection for drought-related phenotypic traits

Using the parental linkage maps developed for the ‘Raleigh x Seville’ population [14], a total of 70 QTL associated with six drought-related traits were identified in three experiments (Table 3). Among them, 46 QTL were mapped on linkage groups for Raleigh, while 24 QTL were found on the Seville map. These QTL were identified on all of the 18 linkage groups with the exception of S6 and S7.

**Table 3:** Quantitative trait loci (QTL) found to have association with drought related traits in a St. Augustinegrass Raleigh x Seville F_1_ population evaluated under greenhouse (GH17 and GH18) and field conditions (SRS18).

Trait	Exp.	QTL	LG	Position (cM)	Interval (cM)	Nearest marker	LOD	Explained variance (%)	Additive effect	K test	Sig. KW
**GC**	**GH17**	**GC-R1.1**	**R1**	**47.38**	**46.20–48.28**	**SNP16248**	**4.67**	**6.3**	**-2.89**	**13.84**	******
	GH18	GC-R1.2	R1	84.22	83.50–84.52	SNP8153	14.98	4.9	27.77	3.14	*
	GH18	GC-R3.1	R3	24.35	23.32–25.05	SNP339	18.14	6.4	11.57		
	GH18	GC-R3.2	R3	42.38	41.04–43.38	SNP2497	14.56	4.7	-10.62		
	GH18	GC-R4.1	R4	22.48	22.19–23.18	SNP40865	13.22	4.2	6.74		
	GH18	GC-R5.1	R5	19.37	13.74–19.97	SNP50815	10.54	3.1	-6.60	6.04	**
	**GH17**	**GC-R5.2**	**R5**	**62.42**	**61.45–64.32**	**SNP24133**	**5.00**	**6.8**	**-2.50**	**4.72**	**
	GH17	GC-R6.1	R6	25.47	23.41–26.07	SNP42539	6.57	9.2	2.95	4.69	**
	**GH18**	**GC-R7.1**	**R7**	**19.69**	**18.84–20.19**	**SNP32695**	**16.05**	**5.3**	**-16.30**		
	GH18	GC-R8.1	R8	69.90	69.70–70.34	SNP7688	19.71	7.0	-14.19		
	GH18	GC-R8.2	R8	80.25	79.18–80.45	SNP40787	13.45	4.3	23.41		
	GH17	GC-R9.1	R9	8.90	4.40–10.04	SNP53760	7.49	10.7	5.32		
	GH17	GC-R9.2	R9	21.59	20.91–25.39	SNP18877	5.79	8.0	-4.42		
	GH18	GC-S2.1	S2	10.90	9.00–14.44	SNP28246	12.14	3.7	-8.00	3.71	*
	**GH17**	**GC-S2.2**	**S2**	**48.20**	**47.48–48.40**	**SNP32244**	**6.17**	**8.6**	**2.99**	**5.89**	**
	GH18	GC-S2.3	S2	77.97	77.73–78.17	SNP6415	11.36	3.4	-15.30	17.4	*******
	GH18	GC-S2.4	S2	85.62	84.96–85.82	SNP4036	12.89	4.0	-11.76		
	GH17	GC-S3.1	S3	33.00	32.69–33.10	SNP13076	4.40	5.9	-2.73	6.48	**
	GH17	GC-S4.1	S4	49.50	49.40–49.90	SNP62050	5.06	6.8	-13.13		
	GH17	GC-S8.1	S8	12.29	12.29–19.29	SNP10828	5.88	7.9	3.05		
	GH17	GC-S8.2	S8	113.48	113.48–114.68	SNP18912	5.63	7.1	-3.72	6.59	**
**LF**	SRS18	LF-R2.1	R2	19.97	11.77–24.57	SNP28862	12.57	7.2	0.18	9.48	****
	SRS18	LF-R3.1	R3	1.10	0–10.4	SNP15095	14.90	8.9	0.24		
	SRS18	LF-R3.2	R3	38.46	37.36–38.89	SNP34457	12.28	7.9	-0.20	7.42	***
	GH17	LF-R4.1	R4	60.03	58.79–63.03	SNP51619	9.45	7.9	0.35		
	GH17	LF-R4.2	R4	70.68	67.31–72.48	SNP51720	16.82	16.5	-0.54	5.44	**
	SRS18	LF-R6.1	R6	50.19	48.39–51.38	SNP56115	15.16	9.1	-0.31	4.56	**
	SRS18	LF-R6.2	R6	93.26	92.76–97.66	SNP36655	12.23	6.7	0.27		
	**SRS18**	**LF-R6.3**	**R6**	**104.60**	**102.78–105.30**	**SNP43183**	**9.57**	**5.1**	**-0.22**		
	**GH18**	**LF-R6.4**	**R6**	**106.25**	**105.34–107.25**	**SNP54118**	**8.50**	**17.1**	**-0.31**	**5.6**	**
	SRS18	LF-R7.1	R7	99.57	98.89–100.37	SNP27871	12.66	7.2	0.33	4.38	**
	GH17	LF-R8.1	R8	96.25	95.95–96.97	SNP14138	10.41	8.9	-0.29		
	SRS18	LF-S1.1	S1	18.64	18.20–19.44	SNP58267	11.87	6.7	0.43	3.67	*
	SRS18	LF-S1.2	S1	50.10	49.48–51.40	SNP45600	10.62	5.8	0.27	3.18	*
	GH17	LF-S2.1	S2	25.44	22.35–26.54	SNP16935	7.53	6.1	-0.31		
	**GH17**	**LF-S2.2**	**S2**	**47.40**	**46.50–48.18**	**SNP28455**	**19.55**	**20.4**	**0.64**	**12.02**	*****
	GH17	LF-S2.3	S2	81.97	81.77–82.66	SNP62262	7.07	5.6	-0.27		
	GH18	LF-S5.1	S5	18.49	15.46–22.19	SNP23249	6.87	13.5	-0.43		
	GH18	LF-S5.2	S5	40.52	40.22–40.72	SNP3910	7.37	14.4	0.36		
**LW**	GH17	LW-R1.1	R1	0.00	0–3.9	SNP22344	7.19	5.5	-0.48	3.82	*
	**GH17**	**LW-R1.2**	**R1**	**11.85**	**11.45–12.56**	**SNP5027**	**8.17**	**6.2**	**0.54**		
	**GH18**	**LW-R1.2**	**R1**	**12.26**	**11.65–12.56**	**SNP5027**	**5.57**	**6.0**	**0.59**		
	GH18	LW-R1.3	R1	41.33	39.96–41.63	SNP41488	6.25	6.8	-0.35		
	**GH17**	**LW-R1.4**	**R1**	**47.58**	**45.80–48.28**	**SNP16248**	**12.99**	**11.0**	**-0.97**	**7.5**	***
	GH17	LW-R2.1	R2	10.77	10.17–11.69	SNP28169	7.43	5.6	-0.59		
	**GH18**	**LW-R5.1**	**R5**	**67.84**	**61.45–69.14**	**SNP2879**	**13.63**	**17.6**	**1.00**		
	**GH18**	**LW-R6.1**	**R6**	**104.50**	**103.38–105.30**	**SNP43183**	**5.36**	**5.8**	**-0.30**		
	**GH17**	**LW-R6.2**	**R6**	**105.44**	**104.50–106.24**	**SNP50810**	**14.52**	**12.9**	**-0.53**	**3.36**	*
	GH18	LW-S3.1	S3	59.00	58.80–59.26	SNP33379	10.18	12.1	-0.69	11.51	*****
	GH17	LW-S5.1	S5	28.51	27.91–31.43	SNP16139	9.91	7.8	-0.76		
	GH17	LW-S5.2	S5	45.23	44.93–45.59	SNP30261	11.44	9.1	0.88		
	GH17	LW-S5.3	S5	61.43	61.14–61.53	SNP31882	7.89	6.1	-0.57		
	GH17	LW-S8.1	S8	46.28	45.88–47.58	SNP18069	10.09	8.0	-0.60	2.72	*
**NDVI**	**SRS18**	**NDVI-R7.1**	**R7**	**15.92**	**14.32–19.54**	**SNP27494**	**50.24**	**7.7**	**58.48**		
	SRS18	NDVI-R7.2	R7	76.29	76.28–76.39	SNP45148	49.19	7.6	-237.47		
	SRS18	NDVI-R7.3	R7	77.73	77.73–78.26	SNP53130	47.80	7.1	233.17		
	SRS18	NDVI-R7.4	R7	98.06	97.96–98.77	SNP19541	47.99	8.1	209.67	5.66	**
	SRS18	NDVI-S2.1	S2	36.40	35.67–37.10	SNP7382	50.39	8.1	63.54		
	SRS18	NDVI-S2.2	S2	41.92	40.32–44.13	SNP32783	47.72	6.4	-85.23	3.26	*
	SRS18	NDVI-S9.1	S9	20.96	20.04–21.86	SNP47240	47.50	6.8	-26.82	16.65	*******
**RWC**	GH18	RWC-R2.1	R2	140.99	139.26–141.29	SNP61871	4.74	13.1	-2.21	3.06	*
	GH17	RWC-R3.1	R3	56.37	55.19–58.47	SNP10789	46.86	8.3	3.60		
	GH17	RWC-R3.2	R3	65.14	64.24–66.57	SNP32849	58.59	13.9	-4.58		
	GH17	RWC-R3.3	R3	133.07	125.70–133.70	SNP56419	41.04	6.2	-2.51		
	**GH17**	**RWC-R6.1**	**R6**	**105.54**	**100.51–106.24**	**SNP50810**	**36.33**	**4.7**	**-2.45**		
	GH17	RWC-R8.1	R8	30.61	30.14–33.91	SNP57399	30.21	3.5	-2.27		
	GH17	RWC-R9.1	R9	99.00	95.24–102.20	SNP16417	33.91	4.2	2.77		
	GH18	RWC-S3.1	S3	32.14	31.13–32.44	SNP58543	5.39	15.1	-1.86		
**CHC**	GH17	Fv/Fm-R4.1	R4	0.00	0–2.50	SNP59548	4.39	11.2	-2.05	3.7	*
	**GH17**	**Fv/Fm-R4.2**	**R4**	**39.39**	**39.09–39.69**	**SNP47984**	**3.82**	**8.8**	**1.78**	**3.73**	*
	**GH18**	**Fv/Fm-R4.2**	**R4**	**39.39**	**39.29–39.45**	**SNP47984**	**3.62**	**9.3**	**0.83**	**4.22**	**

CHC, chlorophyll content; GC, green cover percent; RWC, leaf relative water content; LW, leaf wilting; LF, leaf firing; NDVI, normalized difference vegetation index; LG, linkage group; LOD, logarithm of odds; K test, the Kruskal-Wallis test statistic; Sig. KW, significance by Kruskal-Wallis analysis. Co-localized QTL were bolded. Significance levels of KW analysis:

*: 0.1,

**: 0.05,

***: 0.01,

****: 0.005,

*****: 0.001,

******: 0.0005,

*******: 0.0001.

Twenty-one QTL for GC were detected on linkage groups R1, R3, R4, R5, R6, R7, R8, R9, S2, S3, S4, S8, with explained variance from 3.1 to 10.7% (Table 3). Ten of them were identified in experiment GH17, while 11 of them were found in experiment GH18. A total of 18 QTL were found to be associated with LF, with nine in SRS18, six in GH17 and three in GH18. These QTL explained between 5.1 and 20.4% of the phenotypic variance, with *LF-S2*.*2* showing the highest explained variance and LOD value (Table 3). Forty QTL for LW were identified in the two greenhouse experiments, which explained 5.36 to 17.6% of the phenotypic variance (Table 3). Six and two QTL for RWC were detected in GH17 and GH18, respectively, with only one of them (*RWC-S3*.*1*) mapping on Seville’s map (Table 3). There were only two QTL (*CHC-R4*.*1* and *CHC-R4*.*2*) mapped for CHC and both of them were located on linkage group R4 (Table 3). A total of seven QTL were identified for NDVI in the field evaluation. These QTL were spread across linkage groups R7, S2 and S9 (Table 3).

**Fig 3:**
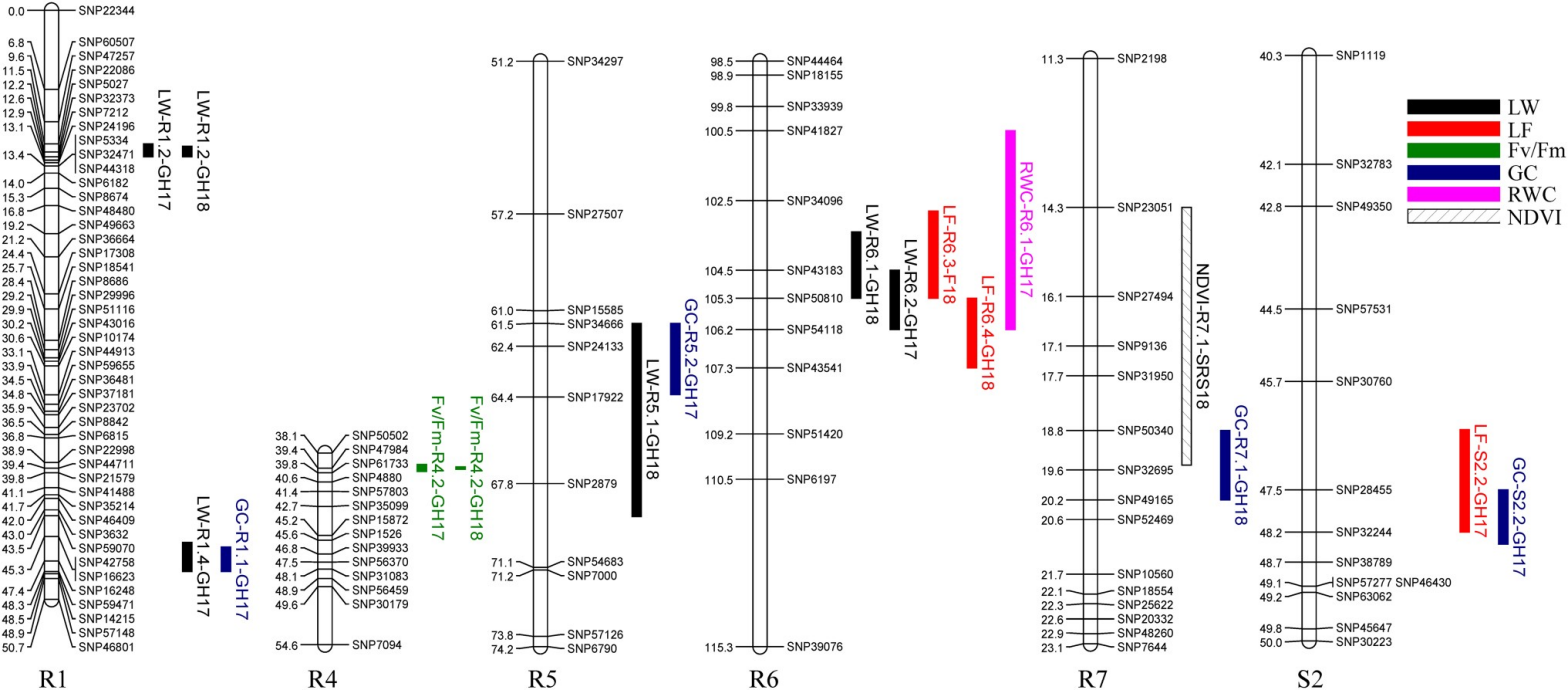
Co-localization of quantitative trait loci (QTL) on linkage groups R1, R4, R5, R6, R7, S2 for drought related traits in St. Augustinegrass Raleigh x Seville F_1_ population evaluated under greenhouse (GH17 and GH18) and field conditions (SRS18). CHC, chlorophyll content; GC, green cover percent; RWC, leaf relative water content; LW, leaf wilting; LF, leaf firing; NDVI, normalized difference vegetation index.

Several QTL for different traits and in different experiments were found to be overlapping in the same genomic regions. QTL *LW-R1*.*2* and *CHC-R4*.*2* were detected in both GH17 and GH18 (Table 3, Fig 3). QTL *GC-R1*.*1* and *LW-R1*.*4* were co-located in region 45.80–48.28 cM of R1, *GC-R5*.*2* and *LW-R5*.*1* overlapped on region 61.45–69.14 cM of R5, *GC-R7*.*1* and *NDVI-R7*.*1* overlapped on R7 (14.32 to 20.19 cM) and *GC-S2*.*2* and *LF-S2*.*2* mapped on S2 (46.50–48.40 cM) (Table 3, Fig 3). In addition, a ‘hotspot’ region was found on R6 from 100.51–107.25 cM, which harbored five QTL (*LF-R6*.*3*, *LF-R6*.*4*, *LW-R6*.*1*, *LW-R6*.*2* and *RWC-R6*.*1*). Among them, the two QTL for LF were identified in GH18 and SRS18, while the QTL for LW was found in GH17 and GH18 (Table 3, Fig 3).

Sequences flanking SNP markers in putative QTL were used to search for candidate genes for drought tolerance. Nine genes were found to be related to drought tolerance in these regions, including ZHD and WRKY transcription factors, ethylene-insensitive protein, cold-responsive protein kinase, OBERON-like protein, light-harvesting complex-like protein (OHP2), Magnesium-chelatase subunit(ChlD), Osmotin-like protein and LRR receptor-like serine/threonine-protein kinase (GSO1) (Table 4).

**Table 4:** Identification of candidate genes within QTL regions associated with drought tolerance. Sequences of SNP were aligned against NCBI database to searching orthologous genes with known function in other species.

Marker	Exp.	QTL	Orthologous gene	Biological function
SNP8153	GH18	GC-R1.2	Ethylene-insensitive protein 2	Ethylene, ABA and stress signaling pathways
SNP2497	GH18	GC-R3.2	ZHD3	Transcription factor
SNP28246	GH18	GC-S2.1	WRKY32	Transcription factor
SNP51619	GH17	LF-R4.1	Cold-responsive protein kinase 1	Stress defense
SNP48621/SNP14138	GH17	LF-R8.1	OBERON-like protein	Root development
SNP31882	GH17	LW-S5.3	Light-harvesting complex-like protein (OHP2)	Photoprotective
SNP47240	SRS18	NDVI-S9.1	Magnesium-chelatase subunit (ChlD)	Chlorophyll biosynthesis
SNP50368	GH17	RWC-R3.2	Osmotin-like protein	Stress defense
SNP58543	GH18	RWC-S3.1	LRR receptor-like serine/threonine-protein kinase (GSO1)	Root development and water transport

## Discussion

QTL analysis has been a primary method used to identify molecular markers linked to drought tolerance related traits, which have the potential to improve selection efficiency for desired traits in breeding [10]. However, molecular markers linked to drought tolerance in turfgrass remain largely unexplored [12]. Genotyping and phenotyping have been the bottlenecks for establishing marker-trait associations of drought stress in turfgrass. This is mostly due to the limited genomic information available for turfgrass species and also to the multigene nature of the genetic control for drought tolerance. In recent years, with the adoption of genotype by sequencing approaches, high density genetic maps have been developed for several turfgrass species [11, 12, 13], including St. Augustinegrass [14]. The genetic maps developed from the ‘Raleigh x Seville’ mapping population contain nine linkage groups for each parent and consist of 2871 SNP markers [14]. These maps provide a powerful foundation for association analysis of traits of interest in St. Augustinegrass. In terms of phenotyping, morphological and physiological traits including GC, LW, LF, NDVI, RWC and CHC have been used as indicators of drought tolerance in the evaluation of turfgrasses [8, 9, 19]. A wide range of phenotypic variance for these traits was found in the ‘Raleigh x Seville’ population evaluated in this study under drought stress conditions under both greenhouse and field conditions (Table 1, Fig 2). However, mean values for each trait were not correlated between different environments, which might be largely due to the enormous environmental difference present between greenhouse and field conditions. Greenhouse experiments provide a controlled, stable environment and reproducible evaluations, but are limited in the size of plots that can be used for evaluation. On the other hand, field experiments reflect ‘real-world’ responses under water deficit stress, but these can also be affected by complicated environmental factors [20]. Given consideration of the environmental difference, both greenhouse and field phenotypic data were collected for QTL analysis in this study, which could enable us to detect drought response QTL in various environments. Significant associations between drought tolerance traits and SNP markers were identified in both greenhouse and field conditions (Table 3, Fig 3). In the future, high-throughput phenotyping platforms might be used to improve the efficiency and precision of drought tolerance evaluation, which could lead to higher reliability in QTL mapping.

A total of 70 QTL associated with six drought-related traits were detected. Among them, 46 QTL were mapped on all nine linkage groups for Raleigh, while 24 QTL were mapped on seven of nine linkage groups for Seville (Table 3). These results suggested that two parents showed different patterns of genetic response to drought stress. Although most of the QTL were found only for a specific trait in one experiment, co-localization of some QTL for different traits and in different experiments was still found in this study (Fig 3). There were significant correlations among most traits under both greenhouse and field conditions. Thus, as expected, QTL for GC overlapped with QTL for LW, as did QTL for LF and NDVI. Likewise, QTL for LF and LW overlapped with QTL for RWC, suggesting that visual rating of LW and LF are reliable approaches for drought evaluation in turfgrass. Responses of LW and LF under drought stress have also been studied on other turfgrass species including zoysiagrass, bermudagrass and tall fescue [21, 22]. Merewitz et al. also found the co-localization of QTL for chlorophyll content, RWC and NDVI in a creeping bentgrass population under drought stress [8]. Identification of QTL associated with multiple traits suggested there might be genes acting epistatically to promote drought tolerance. These findings highlight the value of these regions in gene discovery and function studies. In addition, some co-localized QTL attracted more attention as they were detected in different experiments, specifically under both greenhouse and field conditions. These QTL might be involved in drought response regardless of environmental effects. Beyond that, there were only two QTL identified for chlorophyll content (CHC) in this study, and these QTL were not co-localized with any other traits (Table 3, Fig 3). These results might be due to inadequate genetic variation for CHC among the population for QTL detection.

Through comparative genomic analysis, sequences of SNP markers in St. Augustinegrass were aligned to the genomes of *Setaria italica*, *Sorghum bicolor* and *Oryza sativa* [14]. Several markers closely linked to the QTL identified in this study could be annotated with genes of known function, especially those with functions in drought stress response (Table 4). It is well known that drought resistance in turfgrass may be affected by maintenance of an extensive and deep root system [23]. SNP58543 within QTL *RWC-S3*.*1* had significant sequence match with gene *GSO1*, encoding an LRR receptor-like serine/threonine-protein kinase in *Setaria italica*. In *Arabidopisis*, *GSO1* together with *GSO2* were found to control primary root growth by modulating sucrose response after germination [24]. In addition, QTL *LF-R8*.*1* was matched to gene *OBERON*, which was also reported to regulate root development through the auxin pathway [25]. Thus, these QTL markers could be related to root system development during drought stress. Marker SNP31882 showed high identity with *OHP2*, a gene encoding a light-harvesting complex-like protein, which has been reported to play a photoprotective role within photosystem I in response to light stress [26]. The sequence of SNP47240 was aligned to gene *CHLD* encoding a magnesium-chelatase subunit, which is involved in chlorophyll biosynthesis [27]. It is possible that these markers could respond to drought stress through photosynthetic regulation. However, further research is needed to determine gene function during drought stress in St. Augustinegrass and whether these SNPs could be used for marker-assisted selection.

## Conclusions

Overall, significant phenotypic variance during drought stress was observed among the 115 hybrids in the ‘Raleigh x Seville’ mapping population. A total of 70 QTL associated with six drought tolerance traits were detected, with several QTL co-localizing in the same genomic region. Further candidate gene identification and gene function studies within these QTL regions will contribute to our understanding of the genetic control of drought tolerance in St. Augustinegrass. These QTL have potential value to be used to develop St. Augustinegrass cultivars with improved drought tolerance through marker-assisted breeding.

## Supporting information

S1 Table Variance analysis for chlorophyll fluorescence content (CHC), percent green cover (GC), leaf relative water content (RWC), leaf wilting (LW), leaf firing (LF) and normalized difference vegetation index (NDVI) for a St. Augustinegrass Raleigh x Seville F1 population evaluated for drought response under greenhouse (GH17 and GH18) and field conditions (SRS18). (XLSX)

S2 Table Correlation coefficients between GH17 and GH18 on chlorophyll content (CHC), green cover percent (GC), leaf relative water content (RWC), leaf wilting (LW), and leaf firing (LF). (XLSX)

S1 File Genotypic and phenotypic data used for QTL mapping including genotype loci for hybrids, marker names and positons, and trait values in the Raleigh x Seville population. (XLSX)
